# Persistent nausea burden at peak antiemetic therapy in palliative care: an exploratory study

**DOI:** 10.1186/s12904-026-02122-z

**Published:** 2026-05-01

**Authors:** Paulina Pietsch, Lea Summerer, Ruth Mair, Karolina Müller, Daniel Authier, Wolfgang Herr, Michael Rechenmacher, Annette Schnell

**Affiliations:** 1 Centre for Palliative Care, Internal Medicine III, University Hospital Regensburg, Franz-Josef-Strauß-Allee 11, 93053 Regensburg, Germany; 2 Centre for Clinical Studies, University Hospital Regensburg, Regensburg, Germany; 3 Clinic and Polyclinic for Internal Medicine III, University Hospital Regensburg, Regensburg, Germany

**Keywords:** Palliative care, Nausea, Antiemetic therapy, Biomarker, Cystatin C, Peritoneal carcinomatosis, Ileus, Symptom burden, Personalised medicine, Total pain

## Abstract

**Background:**

Nausea is a common and distressing symptom in palliative care, substantially impairing quality of life. Despite guideline-based antiemetic therapy, a considerable proportion of patients continue to experience substantial nausea burden. Evidence guiding personalised management strategies in this setting remains limited. This study aimed to identify routinely available laboratory markers and clinical factors associated with persistent nausea burden at peak antiemetic therapy.

**Methods:**

In this retrospective exploratory study, 788 admissions to a specialised palliative care unit (2019–2022) were screened, and 223 cases with documented nausea were included. Nausea burden and associated symptom burdens were assessed at the time of “peak antiemetic therapy”, defined as the highest level of antiemetic treatment reached during admission beyond which therapy was not further escalated. Symptom burden was measured using the staff-completed Integrated Palliative Outcome Scale (IPOS). Baseline demographic, clinical, and laboratory variables were assessed at admission. Patients were stratified according to persistent nausea burden (IPOS ≥ 2 vs. < 2). Univariable analyses were performed to identify a core set of associated factors. Baseline variables meeting significance criteria were entered into complete-case binary logistic regression with bootstrap validation (1,000 samples; *N* = 143). Multiple testing was addressed using the Benjamini-Hochberg (BH) procedure.

**Results:**

Persistent nausea burden at peak antiemetic therapy was observed in 33% of patients with nausea. After BH adjustment, vomiting and poor appetite (both IPOS ≥ 2), cystatin C levels, broad-spectrum antiemetic therapy, in-house mortality, ileus, and peritoneal carcinomatosis were significantly associated with persistent nausea burden at peak antiemetic therapy in univariable analyses (BH-adjusted *p* < 0.05). Of these, the baseline variables ileus, peritoneal carcinomatosis, and cystatin C levels constituted the core set for multivariable analysis. In logistic regression, higher cystatin C levels were associated with lower odds of persistent nausea burden (OR = 0.235; 95% CI [0.08–0.47]; BH-adjusted *p* = 0.003), whereas peritoneal carcinomatosis was associated with higher odds (OR = 3.967; 95% CI [1.54–12.29]; BH-adjusted *p* = 0.005).

**Conclusions:**

Persistent nausea burden co-occurred with diverse clinical factors, underscoring its multifactorial nature in advanced disease. Nausea management in palliation remains a major challenge far beyond the application of antiemetics. Prospective studies are warranted.

**Supplementary Information:**

The online version contains supplementary material available at 10.1186/s12904-026-02122-z.

## Background

Nausea is a common and distressing symptom in palliative care [1–3], substantially contributing to suffering, impairing nutrition, and complicating symptom management [4–6]. Reported prevalence of up to 70% varies widely, reflecting differences in populations and assessment methods [4, 5]. In palliative care, nausea is commonly assessed using patient-reported outcome measures that capture both presence and burden [7, 8]. Even mild nausea can significantly diminish quality of life, emphasising the need for effective and individualised control strategies [1]. Moreover, nausea frequently co-occurs with other physical and psychological symptoms, reflecting the multidimensional nature of symptom burden in advanced disease [9].

The aetiology of nausea in palliative care is commonly multifactorial [4, 6]. The evidence base guiding antiemetic choice remains limited, with many recommendations extrapolated from oncology [6]. Although international guidelines advocate a receptor- or mechanism-based approach [6, 10–13], implementation remains challenging given the clinical complexity of nausea in advanced disease [4, 14]. When nausea persists despite standard antiemetic therapy, clinicians frequently rely on empirical escalation strategies, which may delay rapid symptom relief and increase overall burden for a considerable proportion of patients [14].

Research on factors associated with nausea burden has primarily focused on clinical and demographic variables, such as sex, cancer type and opioid exposure [15, 16]. However, these parameters explain interindividual variability only partially, particularly in patients with ongoing nausea despite peak antiemetic treatment.

Biomarkers could complement clinical observation by providing insight into symptom mechanisms or treatment responsiveness [17]. Biomarker-informed approaches have increasingly been discussed in supportive and palliative care, particularly in the context of personalised medicine, with recent reviews highlighting their potential role in symptom prediction and management [18]. Although biological mechanisms, including neurochemical, metabolic, and inflammatory processes, have been proposed as contributors to nausea [19], consistent evidence linking routinely available laboratory parameters to nausea remains limited, particularly outside the context of chemotherapy-induced nausea and vomiting (CINV) [20].

Advancing personalised symptom management consistent with P4 medicine (predictive, preventive, personalised, participatory) requires integrating clinical and biological data to anticipate complex symptom trajectories [21–23]. Therefore, this retrospective exploratory study aimed to identify clinical and biological factors associated with persistent nausea burden at peak antiemetic therapy in palliative care patients, providing a basis for more personalised symptom control.

## Methods

### Study design and setting

This exploratory retrospective study was conducted at the Palliative Care Unit of the University Hospital Regensburg, Germany, covering the period from January 2019 to December 2022.

### Participants and eligibility criteria

Patient cases were considered eligible for univariable analysis when nausea was documented during the palliative care stay or if antiemetic therapy initiated at the referring ward was continued. Documentation of the presence of nausea was derived from physician and nursing notes, admission assessments, daily clinical documentation, and symptom-oriented chart entries. Furthermore, continuation or initiation of antiemetic therapy was considered supportive evidence confirming the presence of nausea.

**Fig. 1:**
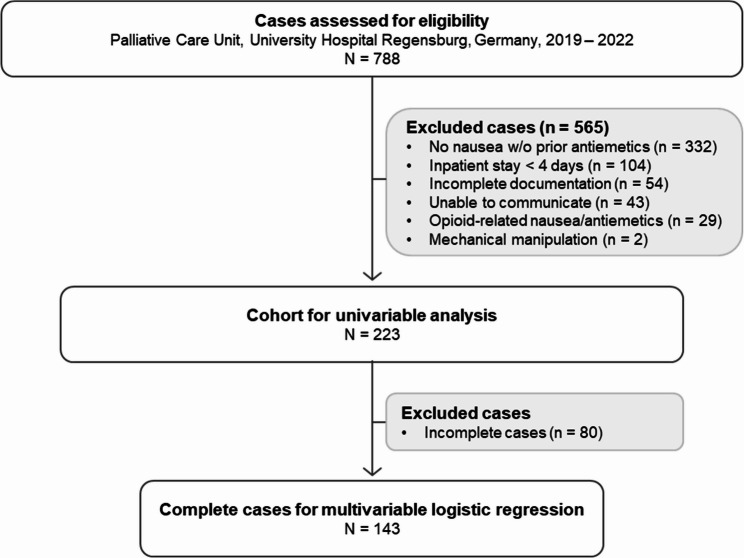
Flow diagram of patient inclusion

Cases were excluded according to predefined exclusion criteria. Those included palliative care stay < 4 days (to ensure ≥ 2 IPOS assessments), incomplete documentation, a persistent non-communicative state, nausea or antiemetic treatment attributable to recent opioid initiation, or vomiting and nausea due to mechanical manipulation (Fig. 1). Descriptive comparisons indicated that the remaining cohort used for univariable analysis was broadly comparable to the total screened population (*N* = 788) across demographic, clinical, medication-related, symptom burden and laboratory characteristics [see Additional file 1 S1].

Of 788 cases assessed for eligibility, 565 were excluded based on predefined criteria. The remaining 223 cases formed the cohort for univariable analysis. Cases with incomplete data for any of the required variables were excluded from multivariable analysis, resulting in 143 complete cases included in the logistic regression.

### Data collection and variables

In accordance with the exploratory design, variable selection followed a priori clinical plausibility, including gastrointestinal, neurological, metabolic, inflammatory and medication-related influences. All variables were extracted retrospectively from electronic medical records, including admission assessments, daily clinical and nursing documentation, oncological records, discharge summaries, and laboratory reports.

Oncological variables included the presence of malignancy, relevant metastatic sites (intracranial, hepatic, and peritoneal involvement), recent anticancer treatments (≤ 1 month), and exposure to immune checkpoint inhibitors. Intracranial metastases comprised cerebral and leptomeningeal involvement and were not differentiated due to limited case numbers.

Clinical conditions were operationalised based on their primary mode of assessment in routine practice. Clinically diagnosed conditions (e.g. ileus, gastritis) were recorded when documented. Mechanical and paralytic ileus were not differentiated due to limited case numbers. Conditions predominantly reflected by laboratory abnormalities (e.g. osseous metastatic disease, infection, renal or hepatic insufficiency) were captured through corresponding laboratory parameters.

Medication variables reflected drugs administered during the palliative care inpatient stay and were selected based on their potential to influence nausea. To avoid misclassification, medications with overlapping indications were classified according to their therapeutic intent. Broad-spectrum antiemetic therapy was defined as treatment with olanzapine or levomepromazine or concurrent use of ≥ 4 narrow-spectrum antiemetic agents, reflecting broad multi-receptor affinity. This operational definition was chosen to capture clinical escalation patterns, in which receptor coverage is broadened when initial agents fail [6, 14]. Antiemetic agents were defined a priori based on clinical practice and included metoclopramide, granisetron, ondansetron, palonosetron, dimenhydrinate, dexamethasone, levomepromazine, olanzapine, aprepitant, midazolam, lorazepam, alizapride, and haloperidol.

Laboratory parameters were obtained from the standardised admission panel and were incorporated comprehensively to allow systematic exploration of routinely available biomarkers. Serum calcium values were albumin-corrected.

Data from the final dying phase were excluded. The final dying phase was defined retrospectively based on clinical documentation indicating that the patient was recognised as “dying” by the treating physician, typically following a multidisciplinary team discussion as part of routine clinical decision-making. This was characterised by a shift in treatment goals towards end-of-life care and primarily comfort-focused treatment (e.g. discontinuation of oral or non-essential medications, adjustment of nursing interventions, initiation of continuous opioid or benzodiazepine infusions when indicated, and inability to participate in symptom assessment) [6].

Variables with very low prevalence (*n* < 10) were excluded at level of univariable screening and therefore not included in any subsequent analysis [24]. A complete list of all evaluated variables, including those excluded from further analyses, is provided in the supplementary data [see Additional file 1 S3]. All extracted data were entered into a structured dataset, checked for internal consistency, and missing values were not imputed.

### Symptom assessment

Symptom burden was assessed using the validated German version of the IPOS [7, 25]; for consistency with international literature, the English IPOS labels are reported. IPOS items were selected a priori when they represented symptoms with a plausible relationship to nausea mechanisms. Accordingly, IPOS items for nausea, vomiting, poor appetite, pain, constipation, anxiousness, and depressed mood were included. To ensure rater consistency and allow inclusion of severely ill patients, only staff-completed IPOS ratings were used, enabling inclusion of a sufficient number of patients.

Symptom assessments referred to the period of “peak antiemetic therapy” to capture refractory symptom burden at the highest level of antiemetic treatment reached during the palliative care inpatient stay. “Peak antiemetic therapy” was defined as the highest level of antiemetic treatment administered to an individual patient during admission, as determined by the treating physicians, beyond which antiemetic therapy was not further escalated. This period therefore reflects the maximum antiemetic therapy considered clinically necessary for the individual patient during admission, rather than the theoretical maximum available. When multiple IPOS assessments were available at this stage, the maximum score was selected.

Persistent nausea burden was defined as an IPOS nausea score ≥ 2 at “peak antiemetic therapy” indicating insufficient symptom control [26]. Other IPOS items assessed at this time point were dichotomised in the same manner (< 2 vs ≥ 2) and analysed as concurrent symptom burden rather than persistent symptoms.

### Statistical analysis

Analyses were conducted using IBM SPSS Statistics 29.0. As almost all continuous variables were non-normally distributed (Shapiro-Wilk *p* < 0.05), a uniform non-parametric approach was applied for univariable analyses: Continuous variables were compared using the Mann-Whitney U-test, categorical variables with the χ² test. Data are presented as medians (IQR = interquartile range) or counts (percentages). Rounding was adapted to the magnitude of values to preserve interpretability across variables with different numeric scales; percentages were rounded to whole numbers and refer to the total available cases per variable. Details on missingness are provided in the supplementary data [see Additional file 1 S1].

In addition, descriptive subgroup analyses were conducted to further characterise patients with persistent nausea burden, including stratification according to escalation to broad-spectrum antiemetic therapy during admission.

To account for multiple testing in univariable and multivariable analyses, p-values were adjusted using the Benjamini-Hochberg (BH) procedure controlling the false discovery rate [27, 28].

To ensure model parsimony and reduce the risk of overfitting given the number of available events, only baseline variables demonstrating statistical significance in univariable analyses after false discovery rate correction (BH-adjusted *p* < 0.05) were included in the core set for subsequent multivariable analysis [24]. Restricting the core set to baseline (pre-outcome) variables ensured temporal precedence in line with recommendations of prediction modelling and allowed priorisation of baseline characteristics as potential predictors of persistent nausea burden at peak antiemetic therapy within an exploratory modelling framework [29]. Variables assessed during admission or contemporaneously with the outcome were not included in multivariable modelling and were interpreted as associated factors rather than potential predictors.

The resulting core set was then entered into a complete-case multivariable binary logistic regression using the enter method. Due to missing values in covariates, a complete-case approach was applied for the regression analysis, avoiding additional assumptions required for imputation-based methods [30]. Descriptive comparisons suggested no relevant differences between the complete-case cohort used for multivariable analyses and the cohort included in univariable analyses regarding demographic, clinical, medication-related, symptom burden, and laboratory characteristics [see Additional file 1 S1].

Statistical outliers were identified using casewise diagnostics based on studentised residuals exceeding ± 2 standard deviations and removed prior to final regression analysis. Multicollinearity among variables entered into multivariable analyses was assessed using pairwise correlation matrices and was not observed. To improve robustness, bootstrap resampling (1,000 samples) was applied. ORs and CIs were rounded to two decimal places. Model fit was evaluated using the omnibus test of model coefficients, Nagelkerkes R², the Hosmer-Lemeshow test, and overall classification accuracy.

## Results

### Patient inclusion and cohort characteristics

A total of 788 cases were screened for eligibility. After application of predefined exclusion criteria, 223 cases (28%) formed the cohort for univariable analysis. Of these, 143 complete cases were available for multivariable analysis (Fig. 1).

Within the univariable cohort (Table 1), persistent nausea burden (IPOS ≥ 2) at peak antiemetic therapy was observed in 33% (*n* = 73). Broad-spectrum antiemetic therapy during admission was administered in 38% (*n* = 84). The median age was 63 years (IQR 53–72), and 46% (*n* = 102) were female. The median length of stay was 14 days (IQR 9–21) and in-hospital mortality occurred in 52% (*n* = 116) of cases. Malignant disease was present in 98% (*n* = 218) of the cohort.

**Table 1:** Characteristics of the cohort for univariable analysis

Variable	Total	No persistent nausea burden	Persistent nausea burden
*Categorical*	*N* = 223 *n (%)*	*N* = 150 *n (%)*	*N* = 73 *n (%)*
Persistent nausea burden at peak antiemetic therapy	73 (33%)	0 (0%)	73 (100%)
Female sex	102 (46%)	64 (43%)	38 (52%)
Male sex	121 (54%)	86 (57%)	35 (48%)
Broad-spectrum antiemetic therapy	84 (38%)	46 (31%)	38 (52%)
In-hospital mortality	116 (52%)	67 (45%)	49 (67%)
Malignant disease	218 (98%)	147 (98%)	71 (97%)
*Continuous [unit]*	*median (IQR)*	*median (IQR)*	*median (IQR)*
Age [years]	63 (53–72)	65 (55–73)	63 (51–72)
Length of in-patient stay [days]	14 (9–21)	13 (9–20)	15 (9–22)

Percentages are rounded to whole numbers and refer to the total available cases per variable. The complete list of evaluated variables and additional descriptive data are provided in the supplementary data [see Additional file 1 S1]

*IQR*  interquartile range

Age, length of stay, and proportion of malignant disease were comparable between patients with and without persistent nausea burden. Although not statistically significant, women were numerically more represented among patients with persistent nausea burden (52% vs. 43%), whereas men were more frequently represented in the non-persistent group (57% vs. 48%). In-hospital mortality was descriptively higher among patients with persistent nausea burden (67% vs. 45%).

Notably, although broad-spectrum antiemetic therapy was more common among patients with persistent nausea burden (52% vs. 31%), nearly half of patients with persistent nausea burden (48%) had not received broad-spectrum antiemetic therapy during admission.

To further explore this finding, patients with persistent nausea burden were descriptively stratified according to escalation to broad-spectrum antiemetic therapy (Table 2, Additional File 1 S2). Within this subgroup, those receiving broad-spectrum antiemetic therapy had a longer median length of stay (18.5 vs. 12 days) and numerically higher proportions of hepatic metastases (39% vs. 20%) and peritoneal carcinomatosis (45% vs. 34%). Vomiting burden was also descriptively higher in this subgroup, whereas age, sex distribution and in-hospital mortality were comparable between patients with and without broad-spectrum antiemetic therapy.

**Table 2:** Characteristics of the persistent nausea burden subgroup

Variable	Total	No broad-spectrum antiemetic therapy	Broad-spectrum antiemetic therapy
*Categorical*	*N* = 73 *n (%)*	*N* = 35 *n (%)*	*N* = 38 *n (%)*
Female sex	38 (52%)	18 (51%)	20 (53%)
Male sex	35 (48%)	17 (49%)	18 (57%)
In-hospital mortality	49 (67%)	23 (66%)	26 (68%)
Malignant disease	71 (97%)	34 (97%)	37 (97%)
*Continuous [unit]*	*median (IQR)*	*median (IQR)*	*median (IQR)*
Age [years]	63 (51–72)	62 (50–73)	63 (53–71)
Length of in-patient stay [days]	15 (9–22)	12 (8–17)	19 (12–24)

Percentages are rounded to whole numbers and refer to the total available cases per variable. The complete list of evaluated variables and additional descriptive data are provided in the supplementary data [see Additional file 1 S2]

*IQR*  interquartile range

### Clinical and symptom-related differences in univariable analysis

In univariable analyses, patients with persistent nausea burden showed a markedly higher concurrent symptom burden at the time of peak antiemetic therapy (Table 3). Based on unadjusted p-values, persistent nausea burden was more frequent among patients with vomiting (*p* < 0.001), poor appetite (*p* < 0.001), constipation (*p* = 0.009), depressed mood (*p* = 0.020), anxiousness (*p* = 0.026), and pain (*p* = 0.031) burden (all IPOS ≥ 2).

**Table 3:** Univariable analysis and core set definition

Variable		*p*-value	Adjusted *p*-value	No persistent nausea burden	Persistent nausea burden
*Categorical*	*Timing of assessment*	*χ²-test*	*Benjamini-* *Hochberg*	*N* = 150 *n (%)*	*N* = 73 *n (%)*
Vomitingª	peak nausea burden	< 0.001	0.0138	5 (3%)	39 (54%)
Poor appetiteª	peak nausea burden	< 0.001	0.0138	99 (70%)	69 (95%)
Ileus^b^	**baseline**	**< 0.001**	**0.0138**	**12 (8%)**	**18 (25%)**
Peritoneal carcinomatosis	**baseline**	**< 0.001**	**0.0138**	**28 (19%)**	**29 (40%)**
Broad-spectrum antiemetic therapy	during inpatient stay	0.002	0.0183	46 (31%)	38 (52%)
In-hospital mortality	death	0.002	0.0183	67 (45%)	49 (67%)
Constipation^a^	peak nausea burden	0.009	0.0619	75 (50%)	50 (68%)
Benzodiazepines	during inpatient stay	0.02	0.11	65 (44%)	44 (60%)
Depressed mood^a^	peak nausea burden	0.02	0.11	111 (83%)	67 (94%)
Anxiousness^a^	peak nausea burden	0.026	0.1238	122 (88%)	68 (97%)
Pain^a^	peak nausea burden	0.031	0.1312	73 (49%)	47 (64%)
Pantoprazole	during inpatient stay	0.035	0.1375	137 (91%)	72 (99%)
*Continuous [unit]*	*Timing of assessment*	*Mann-* *Whitney-U*	*Benjamini-* *Hochberg*	*median (IQR)*	*median (IQR)*
Cystatin C [mg/L]	**baseline**	**0.004**	**0.0314**	**1.37 (1.06–1.88)**	**1.06 (0.87–1.49)**
Eosinophils [/nL]	baseline	0.027	0.1238	0.05 (0.01–0.12)	0.09 (0.03–0.22)

Percentages are rounded to whole numbers and refer to the total available cases per variable. Variables included in the core set for logistic regression (BH-adjusted *p* < 0.05 + baseline variable) are shown in bold

The complete list of evaluated variables and additional descriptive data are provided in the supplementary data [see Additional file 1 S3]

*IQR* interquartile range

(a) Integrated Palliative care Outcome Scale (IPOS) items were dichotomised as < 2 vs. ≥ 2

(b) Ileus includes paralytical and mechanical ileus

Beyond symptom clustering, persistent nausea burden was associated with markers of gastrointestinal involvement, including ileus and peritoneal carcinomatosis (both *p* < 0.001), and was also linked to in-hospital mortality (*p* = 0.002). Treatment patterns differed as well: patients with persistent nausea burden more frequently received broad-spectrum antiemetic therapy (*p* = 0.002), benzodiazepines (*p* = 0.020), and pantoprazole (*p* = 0.035). In addition, lower median cystatin C levels (*p* = 0.004) and higher median eosinophil counts (*p* = 0.027) were observed in the persistent burden group.

After adjustment for multiple testing using the BH procedure, vomiting (54% vs. 3%; BH-adjusted *p* = 0.014), poor appetite (95% vs. 70%; BH-adjusted *p* = 0.014), ileus (25% vs. 8%; BH-adjusted *p* = 0.014), peritoneal carcinomatosis (40% vs. 19%; BH-adjusted *p* = 0.014), broad-spectrum antiemetic therapy (52% vs. 31%; BH-adjusted *p* = 0.018), in-hospital mortality (67% vs. 45%; BH-adjusted *p* = 0.018) and lower cystatin C levels (median 1.06 vs. 1.37 mg/L; BH-adjusted *p* = 0.031) remained significantly associated with persistent nausea burden at peak antiemetic therapy (BH-adjusted *p* < 0.05).

Overall, persistent nausea burden was observed more frequently in patients with higher concurrent symptom burden, intensified treatment patterns, several clinical and biological characteristics, and in-hospital mortality.

### Baseline factors and multivariable analysis

Among variables remaining significant after false discovery rate correction, only ileus, peritoneal carcinomatosis, and cystatin C levels were assessed at baseline and therefore constituted the core set for multivariable analysis.

**Fig. 2:**
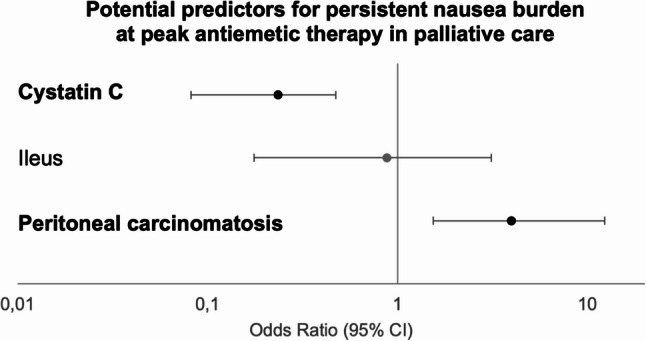
Forest plot of potential predictors for persistent nausea burden at peak antiemetic therapy. Odds Ratios (ORs) derived from binary logistic regression with 1,000 bootstrap samples are shown with 95% bootstrap confidence intervals (CI) on a logarithmic scale. The vertical line represents the no-effect boundary (OR = 1). Black data points indicate variables with adjusted *p* < 0.05; Gray data points indicate non-significant variables

In binary logistic regression with bootstrap resampling (*N* = 143; Fig. 2; Table 4), higher cystatin C levels were associated with lower odds of persistent nausea burden (OR 0.235; 95% CI [0.08–0.47]; BH-adjusted *p* = 0.003). In contrast, peritoneal carcinomatosis was associated with higher odds (OR 3.967; 95% CI [1.54–12.29]; BH-adjusted *p* = 0.005). Ileus did not retain statistical significance in the multivariable analysis.

**Table 4:** Multivariable analysis

Variable [unit]	Odds Ratio	95% CI (bootstrap)	*p*-value (bootstrap)	Adjusted *p*-value	Association
Cystatin C [mg/L]	0.235	0.08–0.47	< 0.001	**0.003**	Lower odds
Ileusª	0.874	0.18–3.11	0.845	0.845	–
Peritoneal carcinomatosis	3.967	1.54–12.29	0.003	**0.005**	Higher odds

Binary logistic regression (complete-case analysis, *N* = 141) with bootstrap correction (1,000 samples). Variables that remained significant after subsequent Benjamini-Hochberg adjustment are shown in bold. Confidence intervals (CI) were rounded to two decimal places

Model fit: omnibus test of model coefficients χ²(3) = 22.083, *p* < 0.001 ; Nagelkerkes R² = 0.208; Hosmer-Lemeshow *p* = 0.290; classification accuracy = 73.8%. Additional model parameters are provided in the supplementary data [see Additional file 1 S4]

(a) Ileus includes paralytical and mechanical ileus

The regression demonstrated a significantly improved fit compared with the null specification (omnibus χ²(3) = 22.083, *p* < 0.001), adequate calibration (Hosmer-Lemeshow test, *p* = 0.29), a Nagelkerkes R² of 0.21, and an overall classification accuracy of 73.8%.

## Discussion

### Principal findings

This exploratory study provides one of the first biomarker-oriented analyses of nausea management in palliative care, examining factors associated with persistent nausea burden at peak antiemetic therapy. One third of patients (33%; Table 1) continued to experience relevant nausea burden (IPOS ≥ 2) despite having reached the highest level of antiemetic treatment considered clinically necessary, identifying a clinically meaningful subgroup with ongoing symptom burden despite pharmacological escalation.

In multivariable analysis, the baseline markers cystatin C and peritoneal carcinomatosis were independently associated with persistent nausea burden, suggesting that biological and disease-related factors may play a central role in refractory nausea. Furthermore, several variables, including concurrent symptom burden, in-house mortality and treatment-related factors, were associated with persistent nausea burden in univariable analyses, although not all remained significant after Benjamini Hochberg correction. Nevertheless, given the exploratory nature of this study, we consider them important, as they may indicate patterns of co-occurring symptoms and clinical deterioration.

Taken together, these results suggest that persistent nausea burden may not represent an isolated symptom entity but rather a manifestation of complex and cumulative suffering, particularly in patients approaching the end of life. This interpretation aligns with the observation that palliation of relevant nausea often remains challenging despite pharmacological escalation and appears to extend beyond the mere application of antiemetics.

In line with concepts of P4 medicine [21, 22] and recent conceptual work in biomarker-informed symptom research in palliative care [18], these findings support a more anticipatory and individualised perspective on symptom management.

### Persistent nausea burden despite escalation: identifying a refractory subgroup

The proportion of patients with persistent nausea burden at peak antiemetic therapy warrants particular attention. Broad-spectrum antiemetic therapy was more frequently administered in this group, supporting the clinical plausibility that treatment was intensified in response to ongoing symptoms. Those patients receiving broad-spectrum antiemetic therapy appeared to represent a more complex disease constellation, characterised by higher proportions of hepatic metastases, peritoneal carcinomatosis and longer hospitalisation, whereas demographic features and mortality were comparable to those patients with narrow-spectrum antiemetics.

Interestingly, nearly half of the patients with persistent nausea burden had not received broad-spectrum therapy during admission, suggesting heterogeneity within this group. For some patients, escalation may not have been clinically indicated, potentially reflecting underlying social, psychological or spiritual factors not captured in this study. For others, overall prognosis, treatment goals, or perceived benefit-risk balance may have influenced therapeutic decisions.

Furthermore, in-hospital mortality was significantly higher among patients with persistent nausea burden, suggesting that this group may represent a more vulnerable population with advanced disease trajectories. Taken together, persistent nausea burden at peak antiemetic therapy does not necessarily reflect insufficient guideline adherence, but rather complex clinical constellations in which pharmacological escalation alone does not fully achieve symptom control.

### Nausea, emotional distress and total pain at the end of life

This clinical complexity becomes particularly evident when considered within the concept of total pain [31, 32]. Within this framework and setting, nausea should not be understood solely as a gastrointestinal symptom but as part of a multidimensional burden encompassing physical, psychological, and existential components.

Persistent nausea burden co-occurred with an increased concurrent symptom burden, including vomiting, poor appetite, pain, anxiousness, and depressed mood, suggesting symptom clustering rather than isolated mechanisms. The higher prevalence of benzodiazepine and pantoprazole use may therefore reflect attempts to manage these complex symptom constellations rather than direct causal relationships [33]. In this context, persistent nausea burden may represent a clinical signal of cumulative disease burden rather than simple under-treatment.

Furthermore, emotional distress appears to play a central role within this multidimensional framework. Anxiousness and depressed mood showed strong univariable associations with persistent nausea burden at peak antiemetic therapy (Table 3), indicating a likely bidirectional relationship: psychological distress may amplify nausea perception, while refractory symptoms may exacerbate emotional burden [34, 35]. Although patients with pronounced emotional distress frequently receive medications with potential antiemetic effects, such as benzodiazepines or mirtazapine [33], persistent nausea burden despite these treatment patterns suggests that psychological factors contribute to refractory symptom constellations beyond pharmacological control.

Consequently, non-pharmacological approaches, psychosocial and behavioural interventions may represent underutilised components of nausea management in palliative care and warrant further investigation [36, 37]. Early identification of this patient subgroup could facilitate timely interdisciplinary reassessment and support a more comprehensive, multiprofessional therapeutic approach.

### Cystatin C as an exploratory biomarker

The observed association between cystatin C and persistent nausea burden represents a novel and hypothesis-generating finding. However, the clinical value of cystatin C as a standalone predictor for persistent nausea burden remains unclear. At present, it should be considered a potential marker for further research rather than a basis for clinical decision-making, as no direct clinical implications might currently be derived. Cystatin C is primarily recognised as a marker of renal function but has also been linked to systemic inflammation and prognostic outcomes [38–40]. In the context of other nausea-dominated conditions, cystatin C has been linked to hyperemesis gravidarum and neurobiological processes [41–43].

Interestingly, creatinine did not show a comparable association, and median values remained largely within the normal range [see Additional file 1 S1]. Therefore, it could be hypothesised that non-renal mechanisms underlie these findings. Alternatively, the diagnostic value of creatinine regarding renal insufficiency may be limited in cachectic or sarcopenic patients [44–46], leaving cystatin C levels to reflect potential downstream effects on antiemetic pharmacokinetics and symptom control [47–49].

Patients with persistent nausea burden showed a higher in-hospital mortality, suggesting that this population may have a poorer prognosis overall. Therefore, we considered that the observed difference in baseline cystatin C between groups may simply reflect the shorter prognosis of patients in the persistent nausea group, rather than a direct association. To further explore the relationship between symptom burden, cystatin C, and disease trajectories, we performed an additional exploratory analysis stratifying patients by outcome (discharged vs. deceased). Among patients who were discharged, a higher nausea burden remained significantly associated with lower cystatin C levels. In contrast, this association was observed only by trend in patients who died during admission. These findings suggest that while prognosis may contribute to the observed association, cystatin C levels may still carry independent information [see Additional file 1 S5].

### Gastrointestinal mechanisms: peritoneal carcinomatosis and ileus

Peritoneal carcinomatosis remained robustly associated with persistent nausea burden in multivariable analysis, consistent with previous reports describing complex gastrointestinal symptom patterns in this population [50, 51]. Ileus showed significant associations in univariable analyses but did not retain statistical significance in multivariable analysis, likely reflecting clinical heterogeneity and limited case numbers.

These findings align with current guideline recommendations advocating mechanism-based antiemetic strategies while highlighting the importance of early identification of gastrointestinal risk constellations.

### Methodological limitations

Given the exploratory and retrospective nature of this study, several limitations should be acknowledged. While causal inference is inherently limited in retrospective analyses, the study design enabled hypothesis generation in a clinically relevant setting. The single-centre design reflects real-world clinical practice but may affect generalisability. Although an objective method was used to secure a sufficient sample size, pre-selection of patients with the symptom nausea was not evaluated using patient-oriented outcomes.

Staff-rated IPOS assessments ensured feasibility and consistency, particularly in severely ill patients; however, they may not fully capture subjective symptom experience compared to patient-reported outcomes. In addition, the time interval between baseline assessment and IPOS nausea evaluation was not incorporated into the analysis.

Furthermore, this study was designed with a predefined focus on nausea burden at peak pharmacological treatment intensity. Expanding the analysis to include non-pharmacological interventions would be highly relevant but would have required a fundamentally different methodological framework.

Complete-case multivariable analysis reduced the available sample size (*N* = 143) but avoided additional modelling assumptions required for imputation and ensured interpretability. Due to false discovery rate correction, some relevant variables might have not been captured. Within this exploratory framework, multivariable analysis should be understood as a structured priorisation of baseline factors rather than confirmation of definitive predictive determinants.

Strengths of the study include a systematically assessed real-world palliative care cohort, the temporal separation of baseline and outcome variables, and the integration of routinely available laboratory parameters. Exclusion of the final dying phase reduced bias related to rapidly changing treatment goals, and statistical robustness was enhanced through bootstrap validation.

## Conclusion

Persistent nausea burden at peak antiemetic therapy identifies a heterogenous and clinically vulnerable subgroup in specialised palliative care. Especially when death is approaching, palliation of relevant nausea as part of complex suffering remains a major challenge and is far beyond the application of antiemetics alone. Our findings support a multidimensional understanding of nausea and highlight the need for prospective multidisciplinary approaches to symptom management in palliative care.

## Supplementary Information

Additional File 1: Supplementary Tables S1–5 (.xlsx). Provides detailed descriptive statistics for all assessed variables, cohort comparisons, and complete results of univariable and multivariable analyses

## Data Availability

All relevant data are provided as supplementary data. The datasets generated during the study are not publicly available due to data protection regulations but are available from the corresponding author upon reasonable request.
